# Achieving Exceptional Mechanical Properties of Epoxy Resins at Ultralow Loadings via a 3DGO@TiO_2_ Hybrid Filler

**DOI:** 10.3390/molecules31142489

**Published:** 2026-07-16

**Authors:** Lizhe Liang, Lan Li, Qiyuan Li

**Affiliations:** School of Mechanical Engineering, Guangxi University, Nanning 530004, China; llan0226@163.com (L.L.); 13330890368@163.com (Q.L.)

**Keywords:** epoxy nanocomposites, nano titanium dioxide, three-dimensional graphene, mechanical properties

## Abstract

Epoxy resin (EP) exhibits pronounced intrinsic brittleness arising from the highly crosslinked network formed after curing, thereby restricting its application in load-bearing structures. Although TiO_2_ nanoparticles possess the potential for impact-strength improvement, they are highly prone to aggregation, which compromises stress-transfer efficiency within the composite. To overcome this challenge, a ball-milling strategy is adopted to anchor TiO_2_ nanoparticles onto three-dimensional graphene oxide (3DGO), leading to the successful fabrication of a 3DGO@TiO_2_ hybrid filler. At an ultralow loading of 0.03 wt%, the 3DGO@TiO_2_ epoxy resin composite shows a 221.5% increase in impact strength to 19.55 kJ/m^2^ and 33.53% and 32.34% increases in tensile and flexural strength to 64.32 MPa and 96.17 MPa, respectively, relative to neat EP. Morphological analyses indicate that the 3DGO spatial confinement reduces TiO_2_ aggregate characteristic length by 55.1% from 1123 nm to 504 nm. Molecular dynamics simulations show that the hybrid filler decreases fractional free volume to 17.6%, induces denser matrix packing, and increases the calculated physical interfacial energy to 1023 kcal/mol, which is 2.2 times that of the pure TiO_2_ epoxy resin system. This work confirms that 3DGO simultaneously optimizes nanofiller dispersion and physical confinement, offering a novel strategy for high-performance epoxy composites at ultralow loadings.

## 1. Introduction

Epoxy resin (EP) has been widely used in aerospace, coatings, and structural composites owing to its excellent mechanical strength, chemical resistance, thermal stability, low shrinkage during curing, and processability under various conditions [1,2,3,4,5]. However, the highly crosslinked network formed after curing leads to intrinsic brittleness and poor resistance to crack initiation and propagation, which severely limits its practical application in load-bearing structures [6,7,8].

To overcome these limitations, various toughening strategies have been developed, including the incorporation of rubber particles, thermoplastics [9,10], and nanofillers [11,12]. Among them, titanium dioxide (TiO_2_) nanoparticles have been widely used to improve the brittleness of epoxy resin due to their good rigidity, excellent thermal stability, and low cost [13,14,15,16,17]. However, TiO_2_ nanoparticles possess high surface energy and tend to aggregate, resulting in non-uniform dispersion in the epoxy matrix. This reduces stress-transfer efficiency, creates stress concentration sites, and promotes premature crack initiation. To address this issue, various strategies such as surface organic modification and ultrasonic-assisted dispersion have been widely adopted to improve the compatibility between TiO_2_ and the epoxy matrix [18,19,20,21]. For example, Yang et al. deposited TiO_2_ nanoparticles onto lignin-based epoxy nanospheres via liquid deposition and incorporated 10 wt% of the hybrid into bio-based epoxy, achieving 33% higher tensile strength and 58% higher fracture toughness [22]. Alternatively, Saikia et al. used 1.0 wt% disulfide-grafted TiO_2_ with ultrasonic dispersion, improving fracture toughness by 136%, tensile strength by 36%, flexural strength by 46%, and storage modulus by 155% [23]. Although effective, the grafting-based strategies typically involve covalent bonding and multi-step surface functionalization, which add complexity and may alter filler surface characteristics [24,25,26,27]. Whether a primarily physical architecture, relying mainly on spatial confinement and mechanical dispersion without intentionally introduced covalent interfacial bonds, can reduce the reliance on such surface engineering while yielding effective dispersion and substantial mechanical reinforcement has rarely been systematically clarified.

Meanwhile, three-dimensional graphene-based architectures have been increasingly adopted to reinforce polymers at low filler loadings [28,29,30,31,32]. For example, Min et al. achieved a 58.6% increase in tensile strength with a 3D graphene/MoS_2_ network at 0.2 wt% [33], and Zhang et al. reported a 57.5% improvement in elastic modulus using a 3D RGO/CNTs/MXene framework at ~0.6 wt% [34]. In many reported graphene-based polymer systems, improved dispersion and interfacial compatibility are achieved through chemical functionalization of the network or the secondary filler [35,36]. By contrast, the use of a 3DGO network to disperse rigid oxide nanoparticles through a simple, primarily physical route relying mainly on spatial confinement rather than intentionally created covalent links has rarely been explored. Consequently, elucidating the structural characteristics of such non-covalently engineered 3DGO-based networks and the resulting filler dispersion state and quantifying the level of mechanical performance that can be attained through this spatial-confinement-assisted strategy remain important yet unaddressed challenges.

Herein, we hypothesize that a three-dimensionally interconnected graphene oxide (3DGO) network can act as a physical template to spatially confine TiO_2_ nanoparticles, thereby suppressing their agglomeration mainly through physical confinement and mechanical dispersion, rather than through intentionally introduced covalent bonds. The continuous 3D skeleton is expected to provide physical contact and mechanical interlocking with the epoxy matrix, enabling improved stress transfer through non-covalent interactions. To test this hypothesis, we address two specific questions: (1) How does the incorporation of the 3DGO@TiO_2_ hybrid filler, at varying loadings in the epoxy matrix, influence the spatial dispersion state and agglomeration behavior of TiO_2_ nanoparticles compared to direct TiO_2_ addition? (2) In the absence of detectable new covalent bonding, can the 3DGO skeleton still contribute to mechanical reinforcement, and what fracture surface morphology and failure features are associated with this non-covalent spatial-confinement-assisted structure? To answer these questions, a 3DGO@TiO_2_ hybrid was fabricated by simple ball milling. The structure and dispersion state were characterized by electron microscopy and spectroscopy, while the mechanical performance was evaluated by impact, tensile and flexural tests. Molecular dynamics simulations were further employed to probe the physical interaction characteristics of the composite system. This design is intended to test whether a spatial-confinement-dominated 3DGO network can achieve improved nanofiller dispersion and mechanical reinforcement without intentionally introduced covalent bonding, aiming to provide a new design concept for nanofiller engineering at ultralow loadings.

## 2. Results

### 2.1. Characterization of 3DGO@TiO_2_

**Figure 1:**
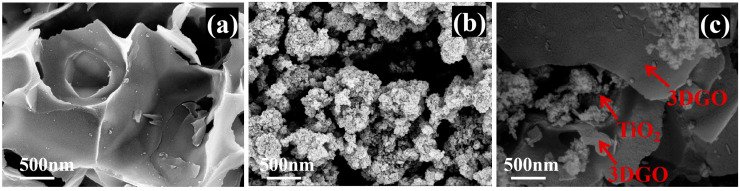
SEM images of (**a**) 3DGO, (**b**) TiO_2_, and (**c**) 3DGO@TiO_2_.

As shown in the Scanning Electron Microscope (SEM) image of the pristine components before ball milling in Figure 1a, 3DGO exhibits a distinct wrinkled sheet-like morphology. The graphene oxide sheets are interconnected and partially overlap to form a three-dimensional framework with visible spatial gaps between adjacent sheets. Overall, 3DGO presents a flower-like wrinkled graphene oxide structure [37]. The Transmission Electron Microscopy (TEM) image in Figure S1 further reveals that thin and wrinkled graphene oxide sheets are interconnected to form a continuous three-dimensional self-supporting framework. This interconnected network structure is beneficial for maintaining the mechanical integrity of the 3DGO framework, while its high specific surface area, as shown in Figure S2, provides anchoring sites for the dispersion of TiO_2_.

In contrast, the pure TiO_2_ particles before ball milling in Figure 1b and Figure S3 are stacked together, forming relatively large TiO_2_ aggregates, indicating obvious aggregation in the observed SEM region. After hybridization via the low-speed ball-milling process, the TiO_2_ aggregates in the resulting hybrid filler, shown in Figure 1c, are distributed on the surface of the graphene oxide framework and in the regions near adjacent graphene oxide sheets. The large-sized TiO_2_ aggregates are clearly reduced, suggesting that the introduction of 3DGO improves the spatial distribution of TiO_2_.

**Figure 2:**
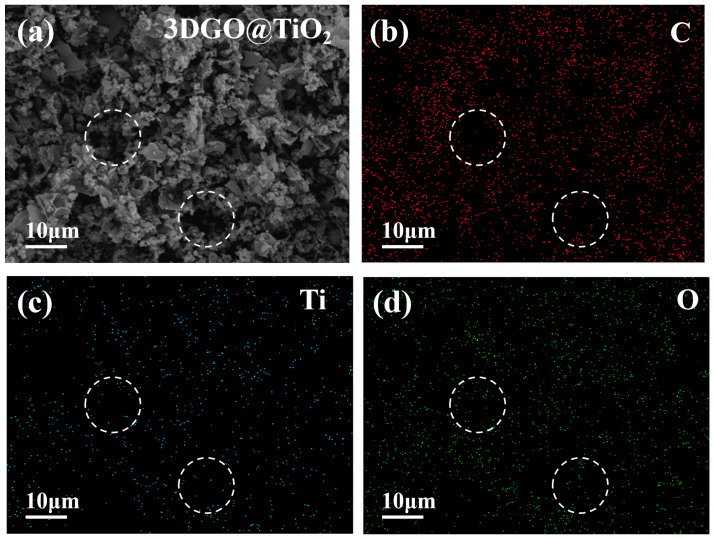
SEM images of (**a**) 3DGO; EDS elemental mappings of 3DGO@TiO_2_: (**b**) C; (**c**) Ti; (**d**) O.

Energy Dispersive X-ray Spectroscopy (EDS) elemental mapping further confirms the hybrid structure of 3DGO@TiO_2_. As shown in Figure 2b–d, the C element is continuously distributed in the observed region, indicating that the 3DGO framework is retained in the hybrid system. To explicitly verify the spatial correlation, white dashed circles are added to highlight specific structural voids in the SEM morphology. These void regions display a corresponding absence of localized elemental signals, confirming the accuracy of the mapping. The Ti and O signals are dispersed within the C-rich region and show good spatial correspondence, confirming that TiO_2_ has been successfully introduced and distributed within the 3DGO structure. This result is consistent with the SEM observations, indicating that TiO_2_ does not mainly exist as independent large-sized aggregates but is partially loaded and dispersed around the 3DGO framework.

**Figure 3:**
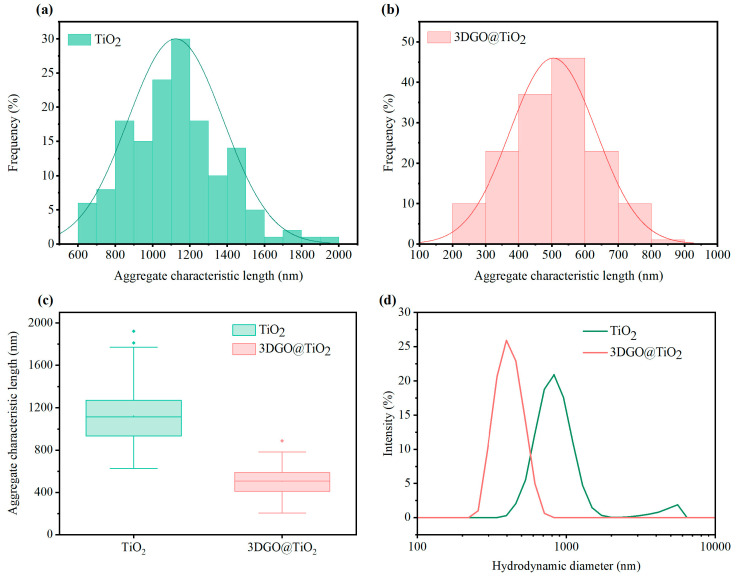
Aggregate size analysis of TiO_2_ and 3DGO@TiO_2_. (**a**,**b**) SEM-derived aggregate characteristic length distributions of TiO_2_ and 3DGO@TiO_2_, respectively; (**c**) corresponding box plots; (**d**) DLS intensity-based hydrodynamic diameter distributions of TiO_2_ and 3DGO@TiO_2_ suspensions in ethanol.

To further quantitatively evaluate the change in TiO_2_ aggregation, the characteristic length of TiO_2_ aggregates was statistically analyzed. As shown in Figure 3a,b, pure TiO_2_ exhibits relatively large aggregates, with the characteristic length mainly concentrated in the range of approximately 1100–1200 nm. After hybridization with 3DGO, the characteristic length distribution of TiO_2_ aggregates in 3DGO@TiO_2_ shifts markedly toward smaller values, mainly concentrating in the range of 400–600 nm. As summarized in Table 1, 150 aggregates were measured for each sample. The average characteristic length of pure TiO_2_ aggregates is 1123 nm, with a maximum value of 1922 nm. In contrast, the average characteristic length of TiO_2_ aggregates in 3DGO@TiO_2_ decreases to 504 nm, and the maximum value decreases to 887 nm, corresponding to reductions of 55.1% and 53.8%, respectively. The box plots further show that the median, interquartile range, and upper range of the characteristic length are all markedly lower for 3DGO@TiO_2_ than for pure TiO_2_ in Figure 3c. These results indicate that the proportion of large-sized TiO_2_ aggregates is reduced and that the overall aggregate characteristic length shifts toward a smaller scale. It indicates that the addition of 3DGO substantially alleviates the observed aggregation of TiO_2_.

DLS was further used to evaluate the hydrodynamic aggregate size of TiO_2_ and 3DGO@TiO_2_ suspensions in ethanol. As shown in Figure 3d and summarized in Table S1, pure TiO_2_ showed a broad intensity-based distribution centered mainly in the submicron range around 0.8 μm, with a weak tail extending toward the micrometer range. This indicates that pristine TiO_2_ existed mainly as secondary aggregates in ethanol, with a small fraction of larger aggregates. In contrast, 3DGO@TiO_2_ exhibited a more concentrated distribution centered at approximately 396 nm, with no obvious micrometer-scale tail under the same dispersion condition. These results suggest that 3DGO reduced the contribution of large hydrodynamic aggregates in the liquid-phase suspension, which is in line with the trend observed from the SEM-based aggregate statistics.

Taken together, the SEM, EDS, TiO_2_ aggregate characteristic length statistics, and DLS results collectively indicate that the introduction of 3DGO markedly reduces the characteristic length of TiO_2_ aggregates and lowers the proportion of large-sized TiO_2_ aggregates. The DLS results further provide suspension-state evidence that 3DGO@TiO_2_ has fewer large hydrodynamic aggregates than pristine TiO_2_ in ethanol, showing the same tendency as the SEM-derived aggregate characteristic length statistics. This result suggests that 3DGO improves the spatial distribution of TiO_2_ within the hybrid structure. Based on the SEM morphology, this improvement may be related to the wrinkled three-dimensional framework of 3DGO, which provides spatial regions for TiO_2_ distribution and partially reduces the direct stacking of TiO_2_ particles, thereby suppressing TiO_2_ aggregation to some extent.

**Figure 4:**
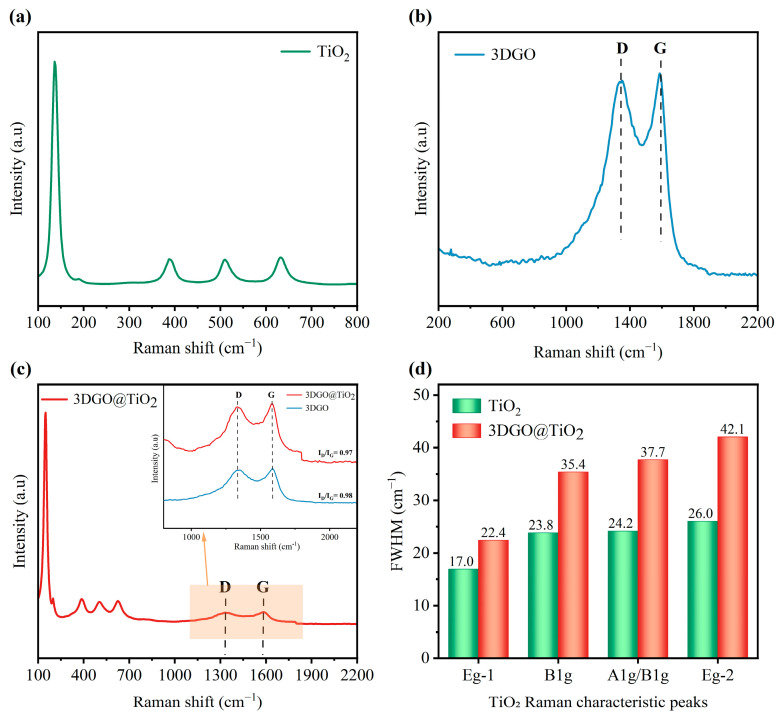
Raman characterization of (**a**) TiO_2_; (**b**) 3DGO; (**c**) 3DGO@TiO_2_; and (**d**) FWHM values of characteristic TiO_2_ Raman peaks.

Raman spectroscopy further reveals the defect characteristics, interfacial interactions, and local microstructure of the 3DGO@TiO_2_ material. As shown in Figure 4a, pure TiO_2_ exhibits distinct Raman peaks at 136, 390, 510, and 632 cm^−1^, corresponding to the characteristic lattice vibration modes of TiO_2_. In Figure 4b, 3DGO shows typical D and G bands at around 1343 and 1586 cm^−1^, which are associated with structural defects in the carbon framework and vibrations of sp^2^-hybridized carbon atoms, respectively [38]. For 3DGO@TiO_2_, both TiO_2_-related Raman peaks and 3DGO-related D/G bands are observed in Figure 4c, confirming the coexistence of TiO_2_ and 3DGO in the hybrid structure and the preservation of the 3DGO framework after hybridization. To rigorously evaluate whether the structural integrity of the carbon skeleton was compromised during physical processing, the localized D and G band regions before and after ball-milling were extracted, normalized, and compared in the inset of Figure 4c. Notably, the I_D_/I_G_ ratio of the 3DGO matrix within the hybrid filler remains nearly unaltered at 0.97, compared to the pristine 3DGO value of 0.98. This negligible variation indicates that the adopted low ball-milling speed successfully prevents graphene oxide degradation and severe lattice disruption, thereby effectively preserving the microstructural integrity of the 3D carbon crystalline framework.

Compared with pure TiO_2_, the TiO_2_ characteristic Raman peaks in 3DGO@TiO_2_ exhibit reduced intensity and broadened peak profiles. As summarized in Figure 4d, the FWHM values of the TiO_2_ Eg-1, B1g, A1g/B1g, and Eg-2 peaks increase from 17.0 cm^−1^, 23.8 cm^−1^, 24.2 cm^−1^, and 26.0 cm^−1^ to 22.4 cm^−1^, 35.4 cm^−1^, 37.7 cm^−1^, and 42.1 cm^−1^, respectively. These results indicate that the introduction of 3DGO may alter the local structural environment of TiO_2_ and increase peak broadening after hybridization. Combined with the SEM, EDS mapping and aggregate-size statistics, the Raman results further support that 3DGO facilitates the dispersion of TiO_2_ through spatial confinement and physical contact.

**Figure 5:**
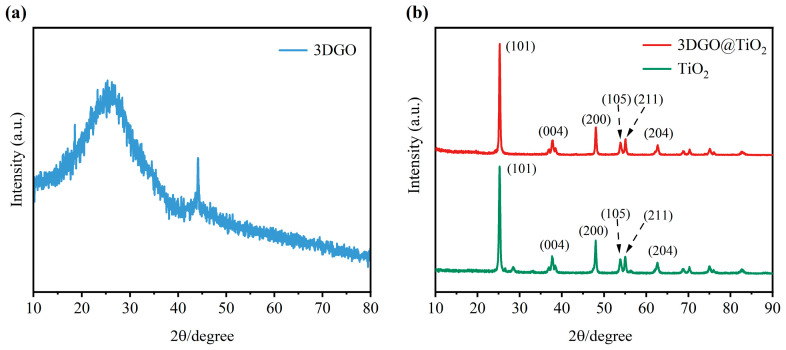
XRD pattern of (**a**) 3DGO; (**b**) TiO_2_ and 3DGO@TiO_2_.

X-ray diffraction (XRD) was used to analyze the crystal structures of TiO_2_, 3DGO, and 3DGO@TiO_2_. As shown in Figure 5a, 3DGO displays a broad peak centered at about 26° and a weak but identifiable peak near 43°. The 26° feature corresponds to the (002) reflection of disordered sp^2^ carbon domains within the graphene oxide framework, indicating a highly disordered layered structure. Its significant broadening reflects poor long-range periodicity and interlayer heterogeneity caused by residual oxygen-containing functional groups. The 43° peak can be associated with the (100) reflection of turbostratic or randomly stacked graphene oxide layers, which further supports the highly defective and disordered nature of the carbon framework. These XRD features are consistent with the Raman, FTIR and XPS results, confirming that the carbon framework should be described as three-dimensional graphene oxide. Figure 5b compares the XRD patterns of pure TiO_2_ and 3DGO@TiO_2_. Pure TiO_2_ displays distinct diffraction peaks at 25.3°, 37.8°, 48.0°, 53.9°, 55.1°, and 62.7°, which match well with the characteristic crystal planes of anatase TiO_2_ (PDF#99-0008), confirming that the TiO_2_ nanoparticles used in this work are predominantly in the anatase crystalline phase [37]. These TiO_2_ peaks are well-preserved in the 3DGO@TiO_2_ hybrid, demonstrating that the crystalline structure of TiO_2_ remains intact after ball milling with the 3DGO framework. The broad diffraction features of 3DGO are barely discernible in the 3DGO@TiO_2_ pattern, primarily because the highly crystalline TiO_2_ nanoparticles exhibit much stronger X-ray scattering, dominating the diffraction intensity and obscuring the weak reflections of the carbon framework. Overall, the XRD results verify the phase composition of the hybrid filler and indicate that the anatase TiO_2_ crystalline structure is retained during the ball-milling-assisted physical hybridization process.

**Figure 6:**
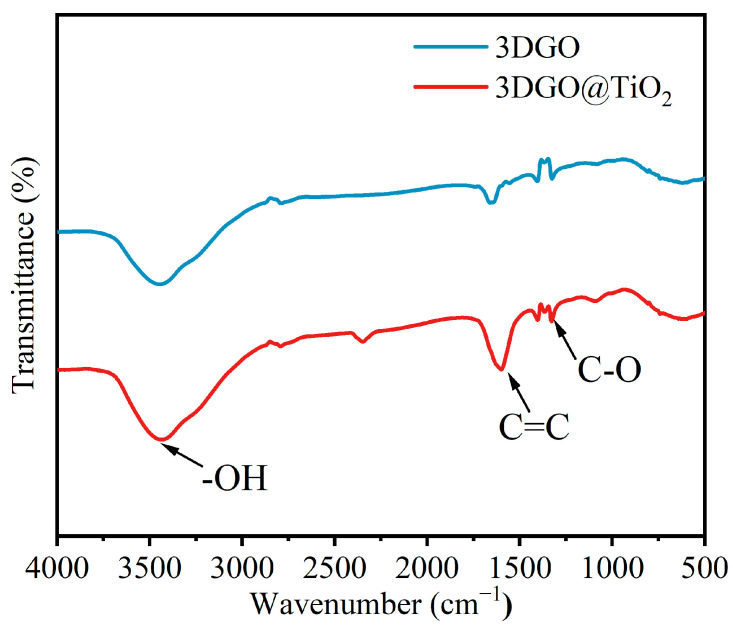
FTIR spectra of 3DGO and 3DGO@TiO_2_.

Fourier Transform Infrared Spectroscopy (FTIR) was used to qualitatively analyze the functional group characteristics of 3DGO and 3DGO@TiO_2_. As shown in Figure 6, both 3DGO and 3DGO@TiO_2_ exhibit a broad absorption band in the range of 3200–3600 cm^−1^, which is assigned to the stretching vibration of -OH groups. In addition, the characteristic absorption peaks associated with the graphitic C=C stretching near 1620 cm^−1^ and the C-O vibrations around 1050 cm^−1^ are clearly retained in the 3DGO@TiO_2_ spectrum. This qualitatively indicates that the GO carbon framework and oxygen-containing functional groups are largely retained during the physical hybridization. No obvious new characteristic absorption peaks are observed after hybridization, indicating that no new covalent bond structure clearly identifiable by FTIR is formed in 3DGO@TiO_2_. This supports the conclusion that the formation of the 3DGO@TiO_2_ composite structure is mainly attributed to physical interactions and non-covalent contact, which is consistent with the ball-milling-assisted physical hybridization process, rather than chemical bonding via newly formed covalent bonds.

**Figure 7:**
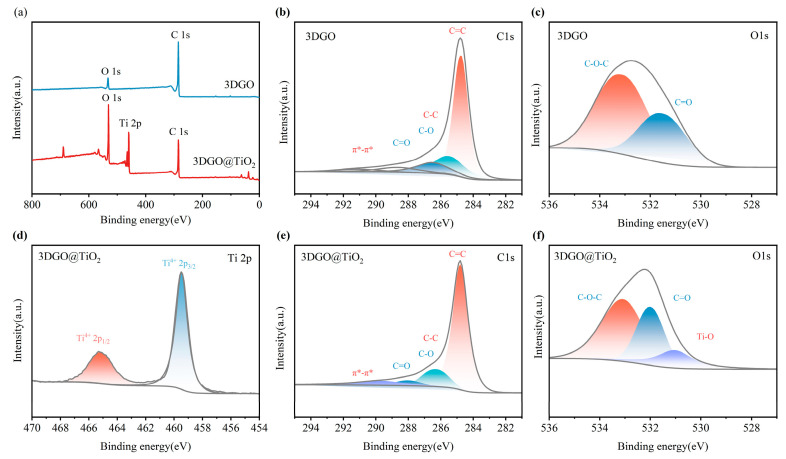
XPS characterization of 3DGO and 3DGO@TiO_2_: (**a**) survey spectra; (**b**,**e**) C 1s spectra; (**c**,**f**) O 1s spectra; (**d**) Ti 2p spectrum of 3DGO@TiO_2_; * represents the π* antibonding orbital.

X-ray Photoelectron Spectroscopy (XPS) was used to analyze the surface composition and chemical states of 3DGO and 3DGO@TiO_2_. As shown in Figure 7a, 3DGO mainly exhibits C 1s and O 1s signals, whereas 3DGO@TiO_2_ shows an additional Ti 2p signal, indicating the presence of TiO_2_ in the 3DGO@TiO_2_ hybrid. The high-resolution Ti 2p spectrum of 3DGO@TiO_2_ in Figure 7d shows two peaks at 459.0 and 464.7 eV, corresponding to Ti^4+^ 2p_3/2_ and Ti^4+^ 2p_1/2_, respectively. The C 1s spectra in Figure 7b,e can be deconvoluted into C=C at 284.8 eV, C-C at 285.5 eV, C-O at 286.2 eV, C=O at 288.0 eV, and a π-π* shake-up satellite at 290.5 eV, indicating that the 3DGO carbon framework and surface oxygen-containing groups are retained after hybridization. In the O 1s spectra shown in Figure 7c,f, the Ti-O component in 3DGO@TiO_2_ further supports the presence of TiO_2_ [39,40]. Together with the FTIR, Raman, and XRD results, the XPS results support the successful formation of the 3DGO@TiO_2_ hybrid structure while showing no clear evidence of new covalent bond formation. Therefore, the formation of the 3DGO@TiO_2_ hybrid structure can be attributed mainly to physical interactions and non-covalent contact between 3DGO and TiO_2_ rather than newly formed covalent bonding. This non-covalent hybridization mode is beneficial for preserving the anatase TiO_2_ crystalline structure and the main chemical features of 3DGO, while facilitating the observed reduction in TiO_2_ aggregation and promoting physical contact through the 3DGO framework.

### 2.2. Mechanical Properties of Composite and Thermal Stability

To examine whether filler sedimentation occurred during curing, cross-sectional SEM-EDS mapping was performed on the top, middle, and bottom regions of the cured 3DGO@TiO_2_/EP composite along the thickness direction. As shown in Figure S4, Ti signals are detected in all three regions, and no obvious Ti-rich accumulation is observed at the bottom side. This result suggests that the 3DGO@TiO_2_ filler was distributed across the thickness direction without significant sedimentation during curing.

**Figure 8:**
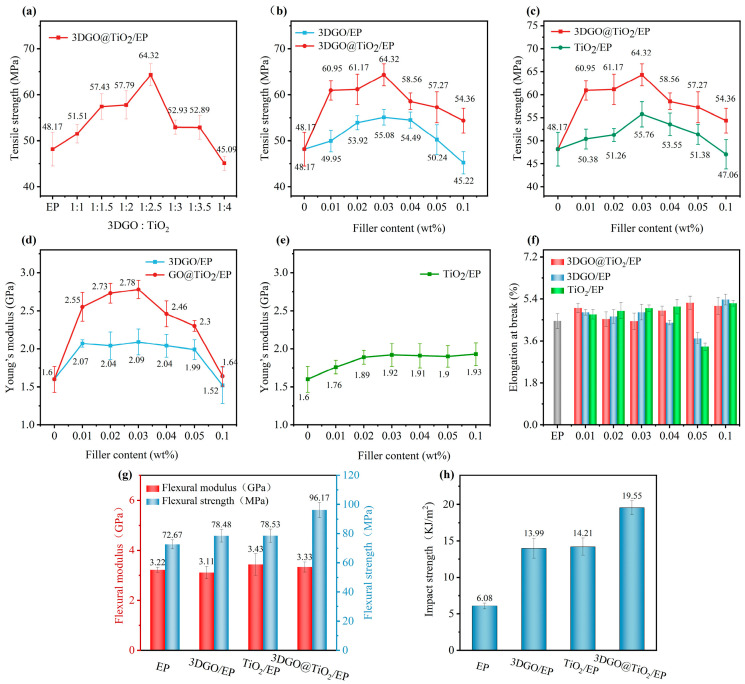
Mechanical properties of EP, 3DGO/EP, TiO_2_/EP and 3DGO@TiO_2_/EP composites: (**a**) tensile strength of 3DGO@TiO_2_/EP with different 3DGO_2_ mass ratios; (**b**,**c**) tensile strength at different filler loadings; (**d**,**e**) Young’s modulus at different filler loadings; (**f**) elongation at break; (**g**) flexural modulus and flexural strength; and (**h**) impact strength.

After confirming the absence of obvious filler sedimentation, the mechanical properties of the epoxy composites were further evaluated. Figure 8 presents the mechanical properties of EP, 3DGO/EP, TiO_2_/EP, and 3DGO@TiO_2_/EP, and the corresponding values are summarized in Table 2. To determine the appropriate mass ratio of 3DGO to TiO_2_ in 3DGO@TiO_2_, the tensile strength of 3DGO@TiO_2_/EP with different 3DGO: TiO_2_ mass ratios was compared. As shown in Figure 8a, the tensile strength initially increases and then decreases with increasing TiO_2_ content, reaching a maximum value of 64.32 MPa at a 3DGO: TiO_2_ mass ratio of 1:2.5, which is 33.53% higher than that of neat EP (48.17 MPa). Therefore, a 3DGO: TiO_2_ mass ratio of 1:2.5 was selected as the experimentally optimized ratio for the subsequent preparation of 3DGO@TiO_2_/EP composites.

Figure 8b,c further compare the tensile properties of different composite systems at various filler contents. The tensile strengths of 3DGO/EP, TiO_2_/EP, and 3DGO@TiO_2_/EP all show an initial increase followed by a decrease, with relatively optimal performance achieved at around 0.03 wt%. The maximum tensile strengths of 3DGO/EP and TiO_2_/EP are 55.08 MPa and 55.76 MPa, respectively, corresponding to increases of 14.3% and 15.8% compared with neat EP. In contrast, 3DGO@TiO_2_/EP reaches a tensile strength of 64.32 MPa, representing an increase of 33.53% compared with neat EP and improvements of 16.8% and 15.4% over 3DGO/EP and TiO_2_/EP, respectively. These results indicate that 3DGO@TiO_2_ provides higher tensile reinforcing performance than either 3DGO or TiO_2_ alone. Combined with the morphological characterization results, the 3DGO framework acts as a spatial support and carrier, reducing direct stacking and aggregation among TiO_2_ particles, thereby improving TiO_2_ dispersion and enabling 3DGO@TiO_2_ to produce a greater improvement in tensile strength in the epoxy matrix.

Figure 8d,e show the Young’s modulus of different EP composites. Compared with neat EP, 3DGO/EP and 3DGO@TiO_2_/EP show higher Young’s modulus, with 3DGO@TiO_2_/EP reaching the maximum value at 0.03 wt%, indicating improved tensile stiffness of the epoxy composites. In contrast, TiO_2_/EP shows only a slight change in Young’s modulus. Figure 8f shows that the elongation at break of the composites changes only slightly after filler incorporation. The values are generally comparable to those of neat EP, indicating that the deformation capability of the epoxy matrix is maintained rather than significantly improved. Figure 8g,h show the flexural properties and impact strength of the EP composites. 3DGO@TiO_2_/EP exhibits the highest flexural strength and impact strength, reaching 96.17 MPa and 19.55 kJ/m^2^, respectively. Its flexural modulus remains comparable to that of neat EP, indicating that the flexural stiffness is maintained. The substantial improvement in impact strength indicates that 3DGO@TiO_2_/EP exhibits a higher energy absorption capacity under impact loading.

Overall, 3DGO@TiO_2_/EP exhibits the best tensile strength, flexural strength, and impact strength among the tested samples, demonstrating that the 3DGO@TiO_2_ hybrid filler effectively improves the measured mechanical properties of EP.

To place the mechanical performance of the present composite in the context of related studies, representative epoxy composites reinforced with graphene-based and hybrid fillers are compared in Table S2. Although some reported systems achieved comparable or higher improvements in specific mechanical properties, the present 3DGO@TiO_2_/EP composite exhibits balanced improvements in tensile strength, flexural strength, and impact strength at an ultralow filler loading of 0.03 wt%. Therefore, the present work is described as a competitive ultralow-loading reinforcement strategy rather than a universally superior system.

**Figure 9:**
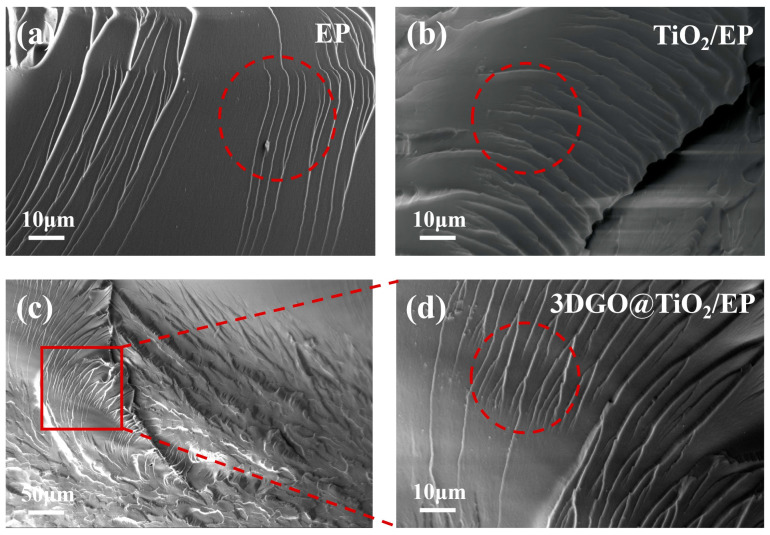
SEM images of fracture surfaces: (**a**) EP; (**b**) TiO_2_/EP; (**c**,**d**) 3DGO@TiO_2_/EP.

Figure 9 shows the fracture-surface SEM morphologies of EP, TiO_2_/EP, and 3DGO@TiO_2_/EP. As shown in Figure 9a, the fracture surface of neat EP is relatively smooth, with regular crack patterns. Specifically, the red dashed circle highlights straight and parallel river-like lines, suggesting a relatively direct fracture path with limited energy dissipation. These features reveal typical brittle fracture characteristics [41,42]. As shown in Figure 9b, after the incorporation of TiO_2_, the fracture surface of TiO_2_/EP becomes rougher. Within the red dashed circle, local undulations and minor crack deflections are visible, suggesting additional fracture-surface roughening and possible energy dissipation during fracture. This is consistent with the improved mechanical properties of TiO_2_/EP compared with neat EP. Notably, the fracture morphology of 3DGO@TiO_2_/EP changes more markedly. As shown in Figure 9c,d, the fracture surface exhibits more wrinkles, tearing traces, and tortuous fracture features, suggesting a more complex fracture process and additional energy-dissipation pathways. This rough and irregular fracture morphology corresponds well to the improved tensile strength, flexural strength, and impact strength of 3DGO@TiO_2_/EP.

Combined with the aforementioned SEM/EDS observations and TiO_2_ aggregate characteristic length statistics of the 3DGO@TiO_2_ filler, the 3DGO framework improves the dispersion of TiO_2_ and reduces the localized aggregation of TiO_2_ particles. As shown in Figure S2, the three-dimensional architecture with a high specific surface area provides abundant physical contact regions, thereby increasing filler–matrix contact opportunities and enabling 3DGO@TiO_2_ to contribute to more tortuous fracture morphology and improved stress distribution under loading, ultimately improving the measured mechanical properties of the composites.

**Figure 10:**
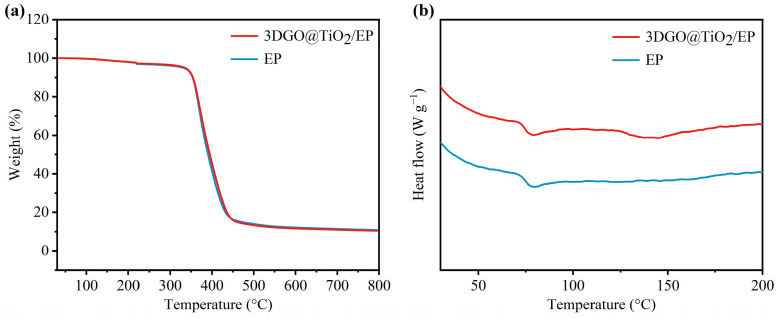
(**a**) TGA curves; (**b**) DSC curves of EP, TiO_2_/EP, and 3DGO@TiO_2_/EP.

Figure 10a shows the TGA curves of neat EP and the 3DGO@TiO_2_/EP composite, and the corresponding thermal parameters are summarized in Table 3. Compared with neat EP, the 3DGO@TiO_2_/EP composite exhibits a slightly delayed thermal degradation process. The T_5_% value increases from 331.4 °C to 335.3 °C, while the temperature at the maximum degradation rate shifts from 370.1 °C to 375.7 °C. However, the final residue at 800 °C does not show an obvious increase, which is reasonable considering the ultralow filler loading [43,44]. To further evaluate the curing state and glass transition behavior, DSC analysis was performed, as shown in Figure 10b. No obvious residual curing exothermic peak is observed for either neat EP or the 3DGO@TiO_2_/EP composite, suggesting that both systems were sufficiently cured under the adopted curing conditions. In addition, the two samples exhibit similar glass transition behavior, indicating that the ultralow loading of 3DGO@TiO_2_ does not significantly alter the final curing state or glass transition behavior of the epoxy matrix. Therefore, the thermal analysis suggests that 3DGO@TiO_2_ slightly delays the main thermal degradation process without significantly changing the curing state of the epoxy resin.

### 2.3. MD Simulation

**Figure 11:**
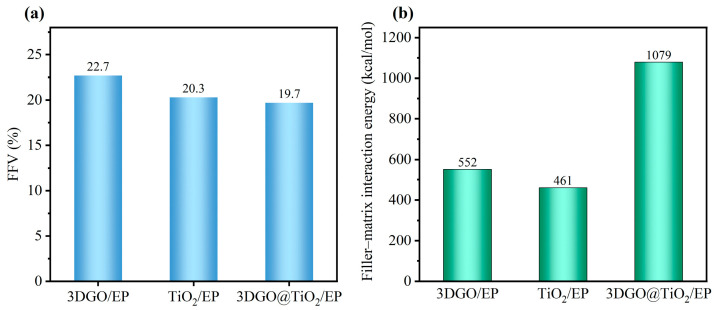
Molecular simulation results of EP composites: (**a**) *FFV*; (**b**) filler–matrix interaction energy.

The *FFV* and filler–matrix interaction energy results are presented in Figure 11 and summarized in Table 4. As shown in Figure 11a, the *FFV* values of 3DGO/EP, TiO_2_/EP, and 3DGO@TiO_2_/EP are 22.7%, 20.31%, and 19.7%, respectively. Among the three systems, 3DGO@TiO_2_/EP exhibits the lowest *FFV*, suggesting that the 3DGO@TiO_2_ hybrid filler more effectively reduces the local free space in the simulated epoxy network and promotes a more compact molecular packing state. This result indicates that the hybrid structure composed of the 3DGO framework and TiO_2_ nanoparticles jointly restricts the mobility of epoxy molecular chains and promotes the formation of a more compact filler/matrix interfacial region.

As shown in Figure 11b, the calculated filler–matrix interaction energies of 3DGO/EP, TiO_2_/EP, and 3DGO@TiO_2_/EP are 552, 461, and 1079 kcal/mol, respectively. Under the same simulation protocol, 3DGO@TiO_2_/EP exhibits the highest interaction energy, which is approximately 2.0 times that of 3DGO/EP and 2.3 times that of TiO_2_/EP. This result suggests that the 3DGO@TiO_2_ hybrid filler provides stronger local interactions with the epoxy matrix than the single-filler systems. Combined with the *FFV* results, the higher interaction energy of 3DGO@TiO_2_/EP supports the formation of a more compact local filler/matrix interfacial region. This trend is consistent with the improved filler dispersion, mechanical performance, fracture morphology, and thermal stability observed experimentally.

## 3. Experimental

### 3.1. Materials and Methods

The 3DGO used in this study was self-prepared by our research group according to the procedure reported in the literature [45], and its preparation method is described in the Supporting Information. The epoxy matrix system consisted of epoxy resin E-51 and curing agent 593 (C_11_H_27_N_3_O_2_), both supplied by Shanghai Autun Chemical Technology Co., Ltd. (Shanghai, China). Nano titanium dioxide (TiO_2_, anatase phase) was obtained from Shanghai McLean Biochemical Technology Co., Ltd. (Shanghai, China). Its primary particle size was 61 ± 9 nm based on high-magnification SEM image statistics, and the BET specific surface area was 49.3 m^2^ g^−1^. Hexadecyl trimethyl ammonium bromide (CTAB) was purchased from Shanghai Aladdin Biochemical Technology Co., Ltd. (Shanghai, China). Detailed physicochemical information on the main components is summarized in Table S3, and the TiO_2_ particle size distribution is provided in Figure S5.

#### 3.1.1. Synthesis of 3DGO@TiO_2_

The 3DGO used in this study was prepared by an autocatalytic method, as shown in Figure S6. On this basis, 3DGO@TiO_2_ nanocomposites were prepared by liquid-phase mixing combined with ball milling. First, 0.5 g of TiO_2_ nanoparticles was dispersed in 160 mL of deionized water, followed by the addition of 40 mL of ethanol and 1 mL of CTAB dispersant. As a surfactant, CTAB generated a large amount of foam during stirring, thereby promoting the interaction between the TiO_2_ nanoparticles and 3DGO [46]. The resulting suspension was sonicated for 30 min to prepare Solution A. Additionally, 0.2 g of 3DGO was ultrasonically dispersed in 20 mL of ethanol to prepare Solution B. Subsequently, Solution B was slowly added to Solution A under magnetic stirring at 80 °C and 900 rpm. After continuous stirring for 2 h to ensure thorough mixing, a homogeneous suspension (Solution C) was obtained. Finally, Solution C was subjected to 24 h of planetary ball milling at 200 rpm to synthesize the 3DGO@TiO_2_ nanocomposite. The resulting product was then collected by vacuum filtration, washed five times, and dried in an oven at 80 °C for 12 h. Compared with hydrothermal/solvothermal routes, this liquid-phase mixing/ball-milling-assisted method does not require sealed high-pressure reaction equipment, although solvent-assisted dispersion, washing, and drying steps are still involved, as summarized in Table S4.

#### 3.1.2. Fabrication of Epoxy Composites

First, the 3DGO@TiO_2_ nanocomposite was dispersed in ethanol at a mass ratio of 1:100 and sonicated for 2 h to obtain a uniform suspension. Subsequently, the suspension was added to E-51 epoxy resin in a specific ratio, with ethanol serving as a temporary diluent to improve the resin’s flowability and promote filler dispersion. After thorough mixing, the mixture was transferred to a ball-milling jar and milled for 12 h at a speed of 200 rpm. During the ball-milling process, the shear forces generated by the rolling and friction of the grinding media effectively disrupted the graphene oxide agglomerates, thereby enhancing the dispersion of 3DGO@TiO_2_ in the epoxy resin matrix [47]; a lower ball-milling speed was chosen to ensure that shear stress dominated the process and to avoid excessive impact forces that could damage the graphene oxide structure [48].

**Figure 12:**
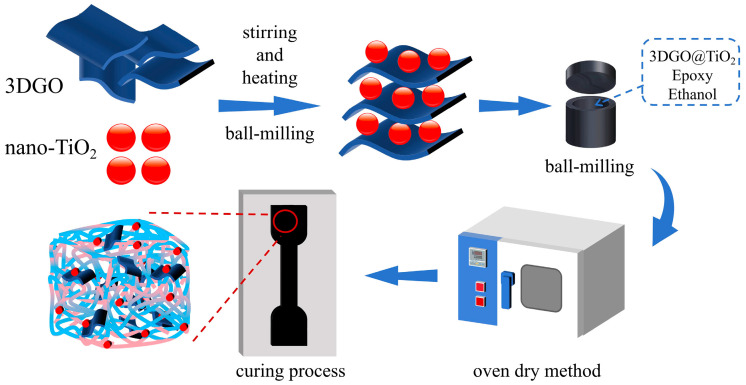
The schematic diagram of 3DGO@TiO_2_ nanocomposite synthesis and the process of embedding 3DGO@TiO_2_ into the epoxy matrix.

After ball milling, the mixture was dried in a vacuum oven at 80 °C for 48 h to remove residual ethanol. Subsequently, curing agent 593 was added to achieve a mass ratio of 5:1 with epoxy resin E-51. After adding the curing agent and mixing uniformly, the mixture was vacuum-degassed at 25 °C for 10 min before being poured into the mold. The mixture was thoroughly homogenized and cured at 80 °C for 2 h, ultimately yielding the 3DGO@TiO_2_/epoxy resin composite. The preparation process is shown in Figure 12.

For comparison, the pure epoxy resin (EP) control sample was prepared using the exact same sequence, including ethanol addition, low-speed ball milling, and subsequent solvent evaporation, to systematically account for any potential effects of mechanical milling and solvent processing on the epoxy molecular weight or curing behavior. Furthermore, the pure 3DGO/epoxy resin (3DGO/EP), pure TiO_2_/epoxy resin (TiO_2_/EP), and 3DGO@TiO_2_/epoxy resin (3DGO@TiO_2_/EP) composites were all prepared using this identical processing protocol.

### 3.2. Mechanical Properties Testing

A microcomputer-controlled electronic universal testing machine (WDW-50KN, Shanghai Zhongyan Instrument Manufacturing Co., Ltd., Shanghai, China) was used to evaluate the tensile and flexural properties of epoxy composites at room temperature (25 °C). Tensile testing was conducted in accordance with ISO 527-2:2012 [49], using a YYU-5/25 electronic extensometer (supplied by Jinan Nake Industry and Trade Co., Ltd., Jinan, China) with a tensile rate of 2 mm/min. The specimens were dumbbell-shaped and polished, with dimensions of 4 mm in width, 4 mm in thickness, and a central effective length of 25 mm. Flexural tests were conducted as three-point flexural tests in accordance with the GB/T 9341-2008 standard [50], with a loading rate of 2 mm/min. The specimens were cast using silicone molds, with dimensions of 80 mm × 10 mm × 4 mm. The resin was vacuum degassed, and the mold was coated with dimethyl silicone oil to facilitate demolding. The support span was set according to the standard span-to-thickness ratio specified in GB/T 9341-2008. Impact testing was performed on notched specimens using an impact testing machine in accordance with the GB/T 1043.1–2008 standard [51]. At least five specimens were tested per group, and the average values were taken for the tensile, flexural, and impact results.

### 3.3. Characterization

The surface morphology and microstructure of 3DGO, TiO_2_, and 3DGO@TiO_2_ were observed using scanning electron microscopy (SEM, Sigma500, Carl Zeiss, Oberkochen, Germany). Cross-sectional SEM-EDS mapping was performed along the thickness direction of the cured 3DGO@TiO_2_/EP composite to examine possible filler sedimentation. The statistical size distribution of TiO_2_ aggregates, evaluating 150 units per sample, was quantified from SEM micrographs using ImageJ v1.54 software, with the maximum Feret’s diameter adopted as the characteristic length. Dynamic light scattering (DLS, Malvern Zetasizer Nano ZS90, Malvern Panalytical, Malvern, UK) was performed to evaluate the intensity-based hydrodynamic aggregate size distributions of TiO_2_ and 3DGO@TiO_2_ suspensions in ethanol. Each sample was measured three times, and the average curve was used for comparison. The bulk phase structure was qualitatively analyzed by X-ray diffraction (XRD, SMARTLAB3KW, Rigaku Corporation, Tokyo, Japan). The molecular structure and chemical composition were investigated using a Fourier transform infrared spectrometer (FTIR, VERTEX 70, Bruker Corporation, Karlsruhe, Germany), while Raman spectroscopy (LabRAM HR Evolution, HORIBA, Kyoto, Japan) allowed qualitative examination of molecular structures. The surface chemical composition and valence states were investigated by X-ray photoelectron spectroscopy (XPS, Thermo Fisher Scientific K-Alpha, Waltham, MA, USA). These characterizations were used to verify the formation of the 3DGO@TiO_2_ hybrid and to clarify whether detectable new covalent bonding was generated during the ball-milling-assisted process. The thermal stability of the generated EP, TiO_2_/EP and G@TiO_2_/EP composites was examined by thermogravimetric analysis (TGA). Differential scanning calorimetry (DSC) was further performed to evaluate the curing state and glass transition behavior of neat EP and 3DGO@TiO_2_/EP composites. Comprehensive experimental details for all characterization methods are provided in Table S5 of the Supporting Information.

### 3.4. Molecular Dynamics Simulation

**Figure 13:**
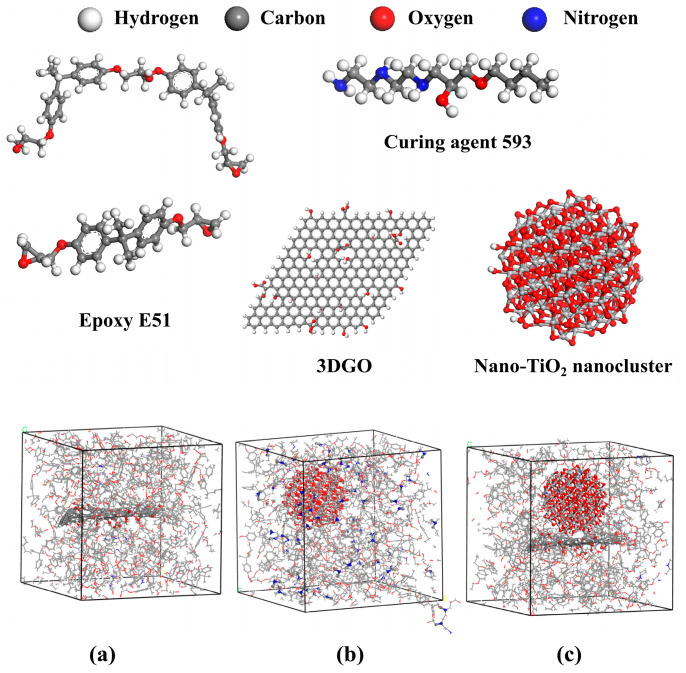
Molecular structures and equilibrated configurations of the MD models: Epoxy E51, curing agent 593, 3DGO, and Nano-TiO_2_ nanocluster are presented: (**a**) 3DGO/EP system; (**b**) TiO_2_/EP system; (**c**) 3DGO@TiO_2_/EP system.

Molecular modeling and simulation were performed using Materials Studio 2020, BIOVIA, Dassault Systèmes, San Diego, CA, USA. The molecular modeling and crosslinking procedures were conducted as follows. First, graphene sheets, TiO_2_ nanoclusters, bisphenol-A epoxy resin molecules, and 593 curing agent molecules were constructed, as shown in Figure 13. The graphene sheet had dimensions of 28.86 Å × 27.43 Å, and the bisphenol-A epoxy resin was represented by two structures with *n* = 0 and *n* = 1. According to the reinforcement composition, three composite systems were established: the 3DGO/EP model containing only graphene, the TiO_2_/EP model containing only a TiO_2_ nanocluster, and the 3DGO@TiO_2_/EP model containing both 3DGO and a TiO_2_ nanocluster. In the 3DGO@TiO_2_/EP model, the TiO_2_ nanocluster was placed above the central region of the graphene sheet to form a 3DGO/TiO_2_ hybrid structure [41].

All initial models were geometrically optimized using the Forcite module in Materials Studio 2020 under the Universal force field, with the maximum number of optimization steps set to 100,000. The Amorphous Cell module was then used to randomly pack the *n* = 0 epoxy molecules, *n* = 1 epoxy molecules, and 593 curing agent molecules into three periodic simulation cells based on the Monte Carlo packing algorithm, obtaining the initial uncrosslinked models. To eliminate unreasonable configurations and stabilize the system density, a 2 ns NPT equilibration was performed at 300 K and 0.1 MPa. Subsequently, five annealing cycles were conducted under the NVT ensemble over the temperature range of 300–900 K with a temperature interval of 50 K, and each stage lasted 10 ps. After annealing, a 2 ns NVT equilibration was further performed at 300 K.

During the crosslinking simulation, epoxy groups and amine groups were defined as reactive sites, and possible reactive atom pairs were gradually searched within a cutoff radius of 3.5–10 Å. A new crosslinking bond was formed when the distance between reactive sites was smaller than the current cutoff radius. After each crosslinking step, geometry optimization and short molecular dynamics relaxation were performed to release local structural stress. This crosslinking–optimization–relaxation process was repeated until the crosslinking degree reached 80%. The 80% crosslinking degree was used as a representative modeling approximation for a highly cured epoxy network, as supported by the DSC results showing no obvious residual curing exothermic peak. Finally, stable crosslinked 3DG/EP, TiO_2_/EP, and G@TiO_2_/EP models were obtained, as shown in Figure 13, and used for subsequent interfacial interaction analysis.

To examine the effects of different fillers on the local molecular packing state of the crosslinked epoxy network and the filler–matrix interaction, the fractional free volume (*FFV*) and filler–matrix interaction energy were calculated based on the equilibrated 3DGO/EP, TiO_2_/EP, and 3DGO@TiO_2_/EP models [52]. It should be noted that these models are simplified representative local interfacial models and are used to compare the relative molecular-level trends under identical simulation conditions, rather than to quantitatively reproduce the full three-dimensional morphology of the experimental 3DGO@TiO_2_/EP composite.

The *FFV* was calculated according to Equation (1):

(1) $$FFV=\frac{V_{f}}{V_{f}+V_{o}}\times 100\%$$

where $V_{f}$ represents the free volume and $V_{o}$ represents the occupied volume. A lower *FFV* generally indicates reduced local free space and a more compact molecular packing state in the simulated epoxy network.

The filler–matrix interaction energy was calculated to compare the local interaction strength between the filler and the epoxy matrix. This parameter has been widely used in molecular dynamics simulations to provide molecular-level insight into filler–polymer interfacial interactions. However, the calculated value represents the cumulative non-bonded interaction within the simulation cell, including van der Waals, electrostatic, and possible hydrogen-bonding contributions. Therefore, it should not be interpreted as an experimentally measurable absolute binding energy. In this work, the interaction energy was used only as a relative indicator for comparing different model systems under the same simulation protocol [53].

The filler–matrix interaction energy was calculated as follows:

(2) $$E_{FMI}=E_{filler}+E_{EP}-E_{total}$$

where $E_{total}$ is the total energy of the composite system, while $E_{filler}$ and $E_{EP}$ are the energies of the isolated filler and epoxy matrix, respectively. A higher $E_{FMI}$ value indicates stronger cumulative filler–matrix interaction under identical model size and calculation conditions.

## 4. Conclusions

In summary, a 3DGO@TiO_2_ hybrid filler was successfully constructed by introducing TiO_2_ nanoparticles onto a self-supporting three-dimensional graphene oxide framework through a liquid-phase mixing/ball-milling-assisted strategy. At an ultralow loading of 0.03 wt%, the 3DGO@TiO_2_/EP composite showed balanced improvements in mechanical properties, with impact strength, tensile strength, and flexural strength increasing by 221.5%, 33.53%, and 32.34%, respectively. The enhancement is mainly attributed to improved filler dispersion and strengthened physical filler–matrix interactions. The 3DGO framework reduced TiO_2_ aggregation and improved the effective contact between the hybrid filler and epoxy matrix. Molecular dynamics results further suggest that 3DGO@TiO_2_ promotes more compact local interfacial packing and stronger non-covalent filler–matrix interactions. Overall, the 3DGO framework and TiO_2_ nanoparticles work synergistically to improve stress transfer and energy dissipation, providing a feasible strategy for interfacial optimization of epoxy composites at ultralow filler loadings.

## Figures and Tables

**Table 1:** Statistical parameters of TiO_2_ aggregate characteristic length.

Samples	N	Mean/nm	SD/nm	Median/nm	Min–Max/nm	CV/%
TiO_2_	150	1123	255	1116	626–1922	22.7
3DGO@TiO_2_	150	504	130	508	204–887	25.8

**Table 2:** Mechanical properties of epoxy and its composites.

Property	EP	3DGO/EP	TiO_2_/EP	3DGO@TiO_2_/EP	Main Change of 3DGO@TiO_2_/EP vs. EP
Tensile strength (MPa)	48.17 ± 3.68	55.08 ± 1.68	55.76 ± 2.75	64.32 ± 2.40	33.53%
Young’s modulus (GPa)	1.60 ± 0.17	2.09 ± 0.17	1.92 ± 0.15	2.78 ± 0.12	73.75%
Elongation at break (%)	4.45 ± 0.32	4.83 ± 0.34	5.00 ± 0.14	4.44 ± 0.36	Comparable
Flexural strength (MPa)	72.67 ± 3.08	78.48 ± 4.34	78.53 ± 4.50	96.17 ± 5.13	32.34%
Flexural modulus (GPa)	3.22 ± 0.10	3.11 ± 0.25	3.43 ± 0.43	3.33 ± 0.20	Comparable
Impact strength (kJ/m^2^)	6.08 ± 0.41	13.99 ± 1.37	14.21 ± 1.18	19.55 ± 0.98	221.55%

**Table 3:** Key TGA parameters of EP and 3DGO@TiO_2_/EP composite.

Samples	T_5_% (°C)	T_max_ (°C)	R800 (°C)	Max. Rate (%·min^−1^)
EP	331.4	370.1	10.84	12.6
3DGO@TiO_2_/EP	335.3	375.7	10.43	11.0

**Table 4:** *FFV* and calculated filler–matrix interaction energy of different composite models.

Property	3DGO/EP	TiO_2_/EP	3DGO@TiO_2_/EP
*FFV* (%)	22.7%	20.3%	19.7%
Filler–matrix interaction energy (kcal/mol)	552	461	1079

## Data Availability

The original contributions presented in this study are included in the article. Further inquiries can be directed to the corresponding author.

## Supplementary Materials

The following supporting information can be downloaded at: https://www.mdpi.com/article/10.3390/molecules31142489/s1, Figure S1. (a,b,d) TEM morphology of 3DGO; corresponding C, N, O elemental mappings of 3DGO for (c) C; (e) N; and (f) O; Figure S2. N_2_ adsorption-desorption isotherm of three-dimensional graphene; Figure S3. SEM images of TiO_2_; Figure S4. Cross-sectional SEM images and Ti elemental mappings of the cured 3DGO@TiO_2_/EP composite at the (a) top, (b) middle and (c) bottom regions; Figure S5. (a) SEM image of TiO_2_ nanoparticles and (b) primary particle size distribution obtained from distinguishable particles in the SEM images. A total of 150 particles were measured, with an average diameter of 61 ± 9 nm; Figure S6. Schematic diagram of the preparation process of three-dimensional graphene; Table S1. DLS derived hydrodynamic size parameters of TiO_2_ and 3DGO@TiO_2_ suspensions in ethanol; Table S2. Comparison of mechanical properties of epoxy composites reinforced with GO-, graphene-, rGO-, and hybrid fillers [36,45,54,55,56,57,58]; Table S3. Physicochemical properties of the main components used in this work; Table S4. Comparison of process conditions and equipment requirements of different preparation strategies; Table S5. Experimental configurations and parameters for characterization instruments.
