# Supplementary material for: Achieving Exceptional Mechanical Properties of Epoxy Resins at Ultralow Loadings via a 3DGO@TiO2 Hybrid Filler
Source: Molecules. 2026 Jul 16;31(14):2489. doi: 10.3390/molecules31142489 (PMC13414806; doi:10.3390/molecules31142489)
Supplement: Supplementary file 1 [file molecules-31-02489-s001.zip › molecules-4394826-supplementary.pdf]

Supporting Information

Achieving Exceptional Mechanical Properties of Epoxy Resins at Ultralow Loadings via a 3DGO@TiO_2 Hybrid Filler

Lizhe Liang, Lan Li and Qiyuan Li*

School of Mechanical Engineering, Guangxi University, Nanning 530004, China;

llan0226@163.com (L.L.); 13330890368@163.com (Q.L.)

* *Correspondence: lianglizhe@gxu.edu.cn*

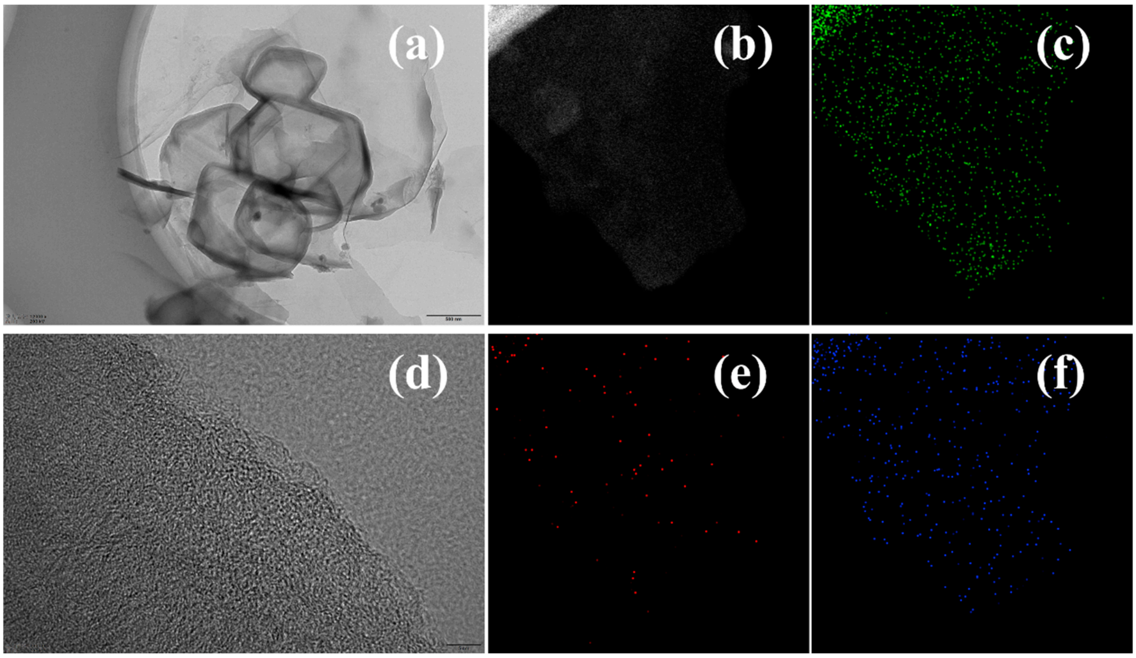

Figure S1. (a,b,d) TEM morphology of 3DGO; corresponding C, N, O elemental mappings of 3DGO for (c) C; (e) N; and (f) O

As shown in Figure S1 (a,b,d), 3DGO exhibits a semi-transparent few-layer sheet-like structure. The graphene sheets are interconnected, curled, and assembled into a continuous three-dimensional wrinkled framework. In Figure S1(a), obvious pores and inter-sheet spaces can be observed, indicating that the sample is not a densely stacked two-dimensional graphene structure but a three-dimensional porous network with spatial support. Figure S1(b) shows that the graphene sheets maintain continuous distribution within the observed region. The thin sheet edges and layered structure in Figure S1(d) further demonstrate the few-layer graphene feature of 3DGO. The elemental mapping images in Figure S1(c,e,f) show that the C signal is continuously distributed along the sheet region, while the N and O signals are dispersed in a dot-like manner, indicating that 3DGO is mainly composed of a continuous carbon framework with surface active sites.

This three-dimensional wrinkled porous framework provides a structural basis for TiO_2 nanoparticle loading. The graphene sheet surfaces, wrinkled edges, and interlayer

pores can serve as attachment regions for TiO_2, providing more dispersion space during the subsequent hybridization process. Compared with densely stacked two-dimensional graphene sheets, the spatial framework of 3DGO can structurally separate nanoparticles and reduce direct contact among TiO_2 particles, thereby suppressing their secondary aggregation. Therefore, 3DGO acts as both a supporting framework and a dispersion carrier in the G@TiO_2 hybrid filler, providing a structural basis for improving the dispersion and interfacial contact of G@TiO_2 in the epoxy matrix.

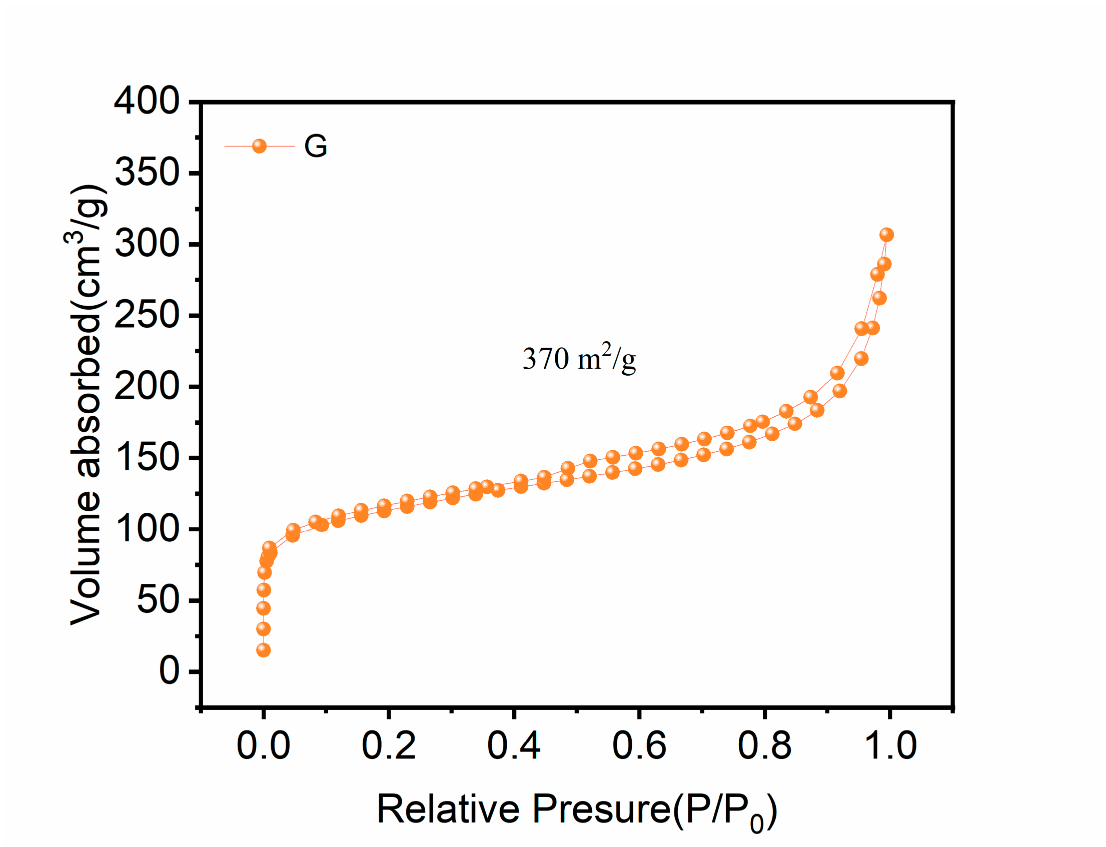

Figure S2. N_2 adsorption-desorption isotherm of three-dimensional graphene.

As shown in Figure S2, the N_2 adsorption－desorption isotherm of 3DGO exhibits typical adsorption characteristics of porous materials. In the low relative pressure region ($P/P_0<0.05$), the adsorption volume increases rapidly, indicating the presence of abundant small-sized pores and strong surface adsorption capacity in 3DGO. As the relative pressure increases, the adsorption volume continues to rise, suggesting that inter-sheet pores and three-dimensional interconnected channels further participate in nitrogen adsorption. In the high relative pressure region ($P/P_0>0.8$), the adsorption volume increases markedly, reflecting the existence of larger pores and open pore structures formed between stacked graphene sheets. The BET results show that the specific surface area of 3DGO is 370 m^2/g, demonstrating its abundant exposed surfaces and porous structure.

This high-specific-surface-area porous framework provides effective attachment sites for TiO_2 nanoparticle loading. The sheet surfaces, wrinkled edges, and pore channels of 3DGO increase the available contact regions between TiO_2 and the graphene framework, allowing TiO_2 to achieve a more sufficient spatial distribution during the hybridization process. Meanwhile, the three-dimensional porous structure provides spatial separation for TiO_2 particles, reducing direct contact between particles and thereby suppressing the secondary aggregation of TiO_2. This result is consistent with the three-dimensional wrinkled sheet-like framework observed in the TEM images, indicating that 3DGO can serve as a porous supporting carrier for TiO_2 nanoparticles.

Table S1. DLS derived hydrodynamic size parameters of TiO_2 and 3DGO@TiO_2 suspensions in ethanol.

Sample	Main peak / nm	D10 / nm	D50 / nm	D90 / nm	Span	Intensity fraction >1000 nm / %
TiO_2	787.5 ± 65.0	549.4 ± 61.1	769.6 ± 11.2	1315.7 ± 393.4	1.00 ± 0.59	22.51 ± 6.96
3DGO@TiO_2	396.1 ± 0.0	291.8 ± 4.2	381.3 ± 12.5	506.1 ± 23.5	0.56 ± 0.03	0

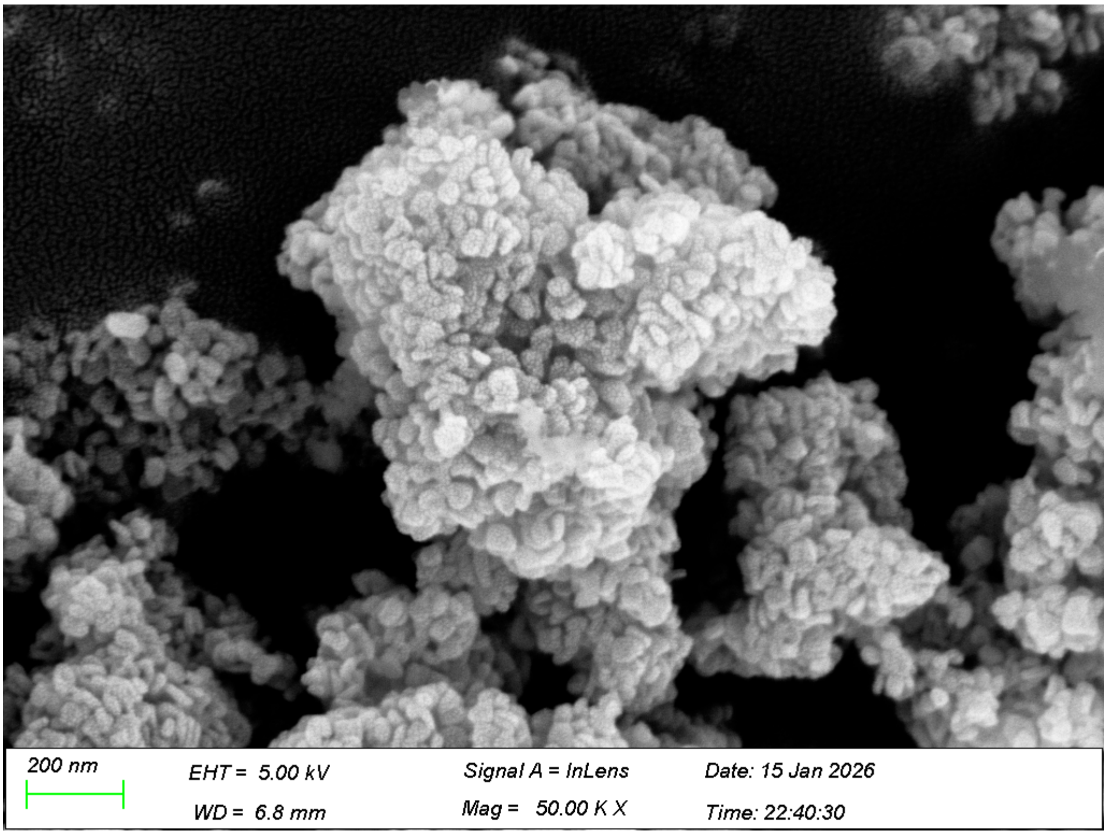

Figure S3. SEM images of TiO_2.

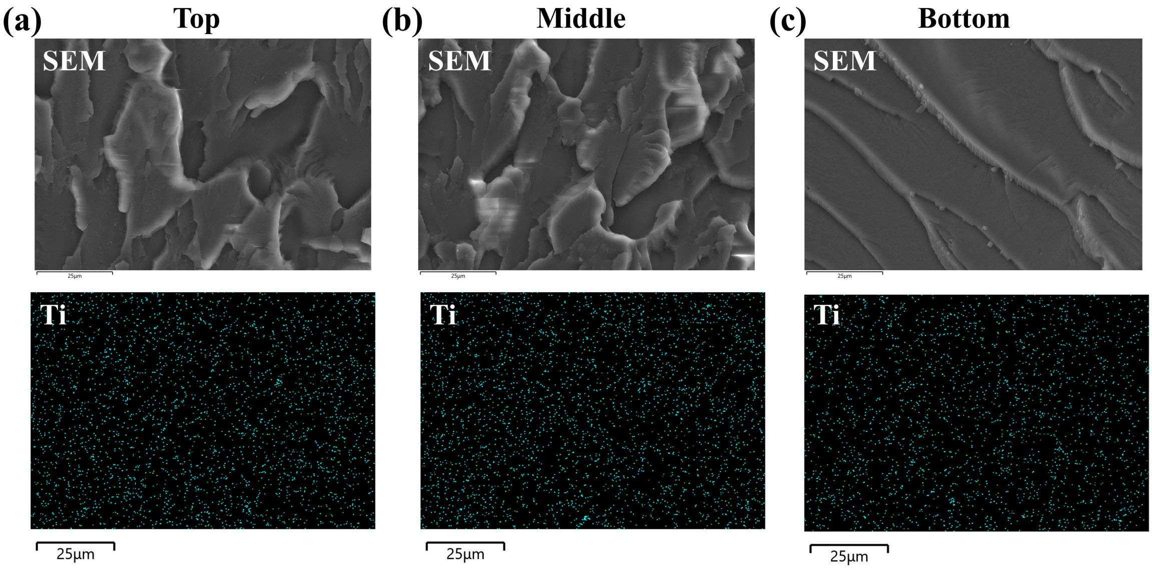

Figure S4. Cross-sectional SEM images and Ti elemental mappings of the cured 3DGO@TiO_2/EP composite at the (a) top, (b) middle and (c) bottom regions.

Table S2. Comparison of mechanical properties of epoxy composites reinforced with GO-, graphene-, rGO-, and hybrid fillers.

Filler system	Filler loading	Modification strategy	Tensile strength increase	Flexural strength increase	Impact strength increase	Reference
TFGE/graphene/EP	<0.03 wt% graphene	Non-covalent π–πF-assisted dispersion	+18.3%	-	+52.1%	Ma et al..[1]
rGO@ZnPB/EP	1.0 wt%	Layered ZnPB functionalized rGO	+17.9%	-	+14.1%	Wang et al.[2]
FGO-E51/EP	0.5 phr GO	Octopus-like GO-based toughener; HTBN grafted onto GO and covalently integrated into epoxy	+25.1%	-	+144.4%	Xue et al..[3]
DF-rGO/EP	0.3 wt% DF-rGO	Dopamine-functionalized reduced graphene oxide; covalent/interfacial reinforcement	-	+73%;	+24%	Jiang et al..[4]
MGO/EP	0.1 wt% MGO	0.1 wt% MGO	+58.3%	+77.1%	+88.0%	Li et al..[5]
GO/CNP/EP	0.125 wt% GO + 0.125 wt% CNP	Hybrid GO/copper nanoparticle reinforcement	+47.51%	+26.83%	-	Mahouri et al..[6]
RGO/MXene-HBPSi/EP	0.7 wt%	Hyperbranched polysiloxane-functionalized RGO/MXene	-	+41.0%	+66.8%	Chen et al..[7]
3DGO@TiO_2/EP	0.03 wt%	Ball-milling-assisted physical hybridization	33.53%	32.34%	221.5%	This work

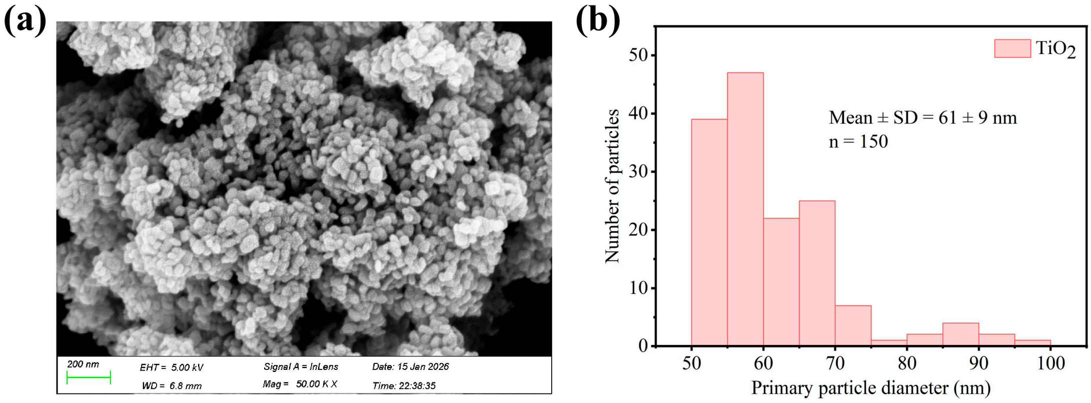

Figure S5. (a) SEM image of TiO_2 nanoparticles and (b) primary particle size distribution obtained from distinguishable particles in the SEM images. A total of 150 particles were measured, with an average diameter of 61 ± 9 nm.

Experiment Section

Preparation process of three-dimensional structured graphene

The three-dimensional graphene oxide (3DGO) powder was prepared through a bottom-up polymerization－pyrolysis route. Typically, 1.0 g of m-phenylenediamine (mPD), 40.0 g of NaCl microcrystals, and 20 mL of anhydrous ethanol were added into a 50 mL agate ball-milling jar containing 35 g of grinding beads. The mixture was ball-milled for 24 h to allow the mPD precursor to uniformly coat the surface of NaCl microcrystals. The obtained mixture was then dried at 80 °C for 12 h to remove ethanol. The dried precursor/NaCl composite powder was transferred into a tube furnace and heated under a N_2 atmosphere. The sample was first maintained at 150 °C for 2 h to complete the in-situ polymerization of mPD on the NaCl surface. Subsequently, the temperature was increased to 1500 °C and maintained for 1 h to induce pyrolysis and graphenization of the polymerized precursor. After cooling to room temperature, the carbonized product was repeatedly washed with deionized water and anhydrous ethanol to remove the NaCl template and residual impurities. Finally, the product was dried in a vacuum oven at 80 °C for 12 h to obtain 3DGO powder.

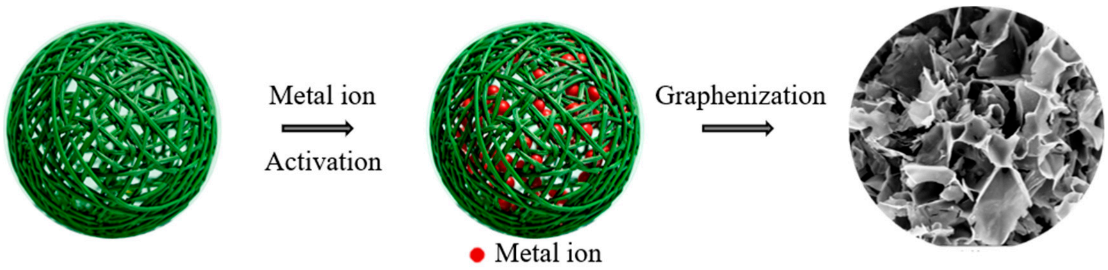

Figure S6. Schematic diagram of the preparation process of three-dimensional graphene.

In this process, NaCl microcrystals served as removable templates, enabling the polymerized carbon precursor to grow on their surface and preventing severe restacking during carbonization. After template removal, the resulting 3DGO powder formed a self-supported porous framework composed of interconnected few-layer graphene sheets, which provided a suitable scaffold for subsequent TiO_2 loading and epoxy composite reinforcement.

Table S3. Physicochemical properties of the main components used in this work.

Component	Description / chemical type	Key parameters	Supplier
E-51 epoxy resin	Bisphenol-A type epoxy resin	EEW: 180 g eq^{-1}; viscosity at 25 °C: 15,000 mPa·s	Shanghai Autun Chemical Technology
593 curing agent	Amine-type curing agent	Active hydrogen equivalent / viscosity: 150 mPa·s	Shanghai Autun Chemical Technology
TiO_2	Titanium dioxide nanoparticles	Anatase phase; primary particle size: 61 ± 9 nm; $D_{10}/D_{50}/D_{90}$: 52/58/70 nm; specific surface area: 49.3 m^2 g^{-1}; purity: 99.7%	Shanghai Aladdin Biochemical Technology
3DGO	Three-dimensional graphene oxide	Specific surface area: 370 m^2 g^{-1}	-
CTAB	Surfactant	Purity: 99.7%	Shanghai Aladdin Biochemical Technology
Ethanol	Solvent	Purity: 99.7%%	Shanghai Aladdin Biochemical Technology

Table S4. Comparison of process conditions and equipment requirements of different preparation strategies.

Preparation strategy	Typical process	Equipment/condition requirement	Cost-related implication
Present ball-milling-assisted method	Dispersion, stirring, drying, ball-milling, washing and drying	Ultrasonication, stirring, drying oven and ball mill; atmospheric pressure	No sealed high-pressure reactor required; equipment complexity is reduced
Hydrothermal/ solvothermal	Solution reaction under elevated temperature and pressure	Sealed autoclave and controlled heating/pressure	Higher equipment and pressure-control requirements
Sol-gel	Precursor hydrolysis/condensation, aging, drying or heat treatment	Controlled precursor reaction and post-treatment	More reaction-control and post-treatment steps
In-situ growth/ deposition	Nanoparticle growth or deposition through solution reactions	Controlled solution reaction, washing and drying	Requires reaction-condition control and post-treatment

Note: This table provides only a qualitative comparison of typical process features among different preparation strategies. The exact conditions may vary depending on the material system and experimental protocol.

Table S5. Experimental configurations and parameters for characterization instruments.

Technique	Instrument (Model)	Key Operating Parameters
Scanning Electron Microscopy (SEM)	ZEISS Sigma500 (Germany)	Accelerating voltage: 5 kV; Working distance: 8.5 mm; Detector: secondary electron; Image analysis: TiO_2 aggregate sizes were quantified using ImageJ v1.54 (150 aggregates per sample, maximum Feret's diameter as characteristic length).
Dynamic Light Scattering (DLS)	Malvern Zetasizer Nano ZS90 (UK)	Dispersant:ethanol dispersant; scattering angle: 90°; measurement temperature: 25 °C; distribution type:intensity-based hydrodynamic diameter distribution; average of three repeated measurements.
X-ray Diffraction (XRD)	Rigaku SMARTLAB3KW (Japan)	Radiation: Cu Kα (λ = 0.15406 nm); Scan range: 10–90°; Scan rate: 10° min^{-1}
Fourier Transform Infrared Spectroscopy (FTIR)	Bruker VERTEX 70 (Germany)	Spectral range: 4000–500 cm^{-1}; Resolution: 4 cm^{-1}
Raman Spectroscopy	HORIBA LabRAM HR Evolution (France)	Laser wavelength: 532 nm; Laser power: 5 mW; Spectral range: 100–3200 cm^{-1}
X-ray Photoelectron Spectroscopy (XPS)	Thermo Fisher Scientific K-Alpha (USA)	X-ray source: Al Kα (photon energy = 1486.6 eV)
Thermogravimetric Analysis / Differential Scanning Calorimetry (TGA/DSC)	METTLER TOLEDO TGA/DSC 1 (Switzerland)	Atmosphere: nitrogen; gas flow rate: 50 mL min^{-1}; heating rate: 10 °C min^{-1}; TGA temperature range: 30–800 °C; the first heating segment within 30–220 °C was used for DSC analysis to evaluate the curing state and glass transition behavior.

1. Ma, T.; Ma, J.; Yang, C.; Zhang, J.; Cheng, J. High-performance, multi-functional and well-dispersed graphene/epoxy nanocomposites via internal antiplasticization and π-πF interactions. Composites Science and Technology 2021, 215, 109008, doi:https://doi.org/10.1016/j.compscitech.2021.109008.
2. Wang, W.; Wang, Z. In-situ preparation of layered zinc N, N′-piperazine (bismethylene phosphonate) functionalizing reduced graphene oxide for epoxy resin with reduced fire hazards and improved thermal/mechanical properties. Composites Part A: Applied Science and Manufacturing 2021, 149, 106588, doi:https://doi.org/10.1016/j.compositesa.2021.106588.
3. Xue, G.; Xue, S.; Sun, M.; Wang, L.; Zhang, X.; Song, C.; Li, J. Biomimetic Octopus-Like Graphene-Based Toughener for Simultaneous Enhancement of Mechanical and Thermal Properties in Epoxy Composites. Polymer Composites 2026, 47, 13106-13120. doi:https://doi.org/10.1002/pc.71147.
4. Jiang, H.; Wang, L.; Shang, Y.; Li, Y. Dopamine-Functionalized Graphene Oxide as Multifunctional Epoxy Modifier: Synergistic Enhancement of Toughness and Thermal Conductivity. Journal of Applied Polymer Science 2025, 142, e57610, doi:https://doi.org/10.1002/app.57610.
5. Li, X.; Yu, B.; Chen, J.; Huo, D.; Liu, J.; Nan, D. Optimized Functionalization of Graphene Oxide for Enhanced Mechanical Properties in Epoxy Resin Composites. Coatings 2024, 14, 609, doi:10.3390/coatings14050609.
6. Mahouri, M.; Parvaneh, V.; Dadrasi, A.; Sabet, G.S. Comprehensive investigation of epoxy/graphene oxide/copper nanocomposites: experimental study and modeling-optimization of mechanical characteristics. Iranian Polymer Journal 2025, 34, 373-385, doi:10.1007/s13726-024-01374-x.
7. Chen, Z.; Zhang, M.; Ren, P.; Lan, Z.; Guo, Z.; Yan, H.; Jin, Y.; Ren, F. Enhanced mechanical and tribological properties of epoxy composites reinforced by novel hyperbranched polysiloxane functionalized graphene/MXene hybrid. Chemical Engineering Journal 2023, 466, 143086, doi:https://doi.org/10.1016/j.cej.2023.143086.
